# Room Temperature Synthesis of Tellurium by Solution Atomic Layer Deposition

**DOI:** 10.1021/acs.cgd.4c00987

**Published:** 2024-09-10

**Authors:** Jessica Willkommen, Amin Bahrami, Nicolas Perez Rodriguez, Angelika Wrzesinska-Lashkova, Yana Vaynzof, Kornelius Nielsch, Sebastian Lehmann

**Affiliations:** † Leibniz Institute for Solid State and Materials Research, Helmholtzstraße 20, 01069 Dresden, Germany; ‡ Chair for Emerging Electronic Technologies, TUD Dresden University of Technology, Nöthnitzer Str. 61, 01187 Dresden, Germany; § Institute of Materials Science, Technische Universität Dresden, Helmholtzstraße 7, 01062 Dresden, Germany

## Abstract

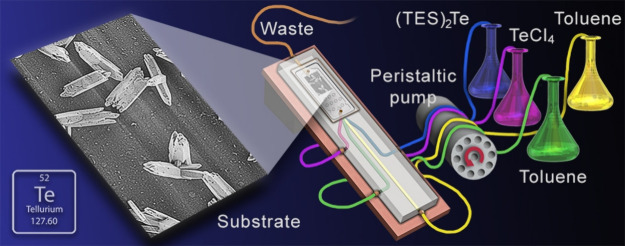

This study demonstrates the deposition of tellurium (Te) on silicon/silicon nitride substrates using solution atomic layer deposition (sALD) at ambient temperature. The process employs tellurium tetrachloride (TeCl_4_) and bis(triethylsilyl)-telluride ((TES)_2_Te) as precursors, with toluene as the solvent. Growth parameters were optimized through systematic variation of the pulse and purge times. Morphological characterization via scanning and transmission electron microscopy revealed needle-like crystallites, while X-ray diffractometry confirmed the crystalline nature of the deposited Te. Increasing the number of deposition cycles resulted in larger Te crystallites and enhanced substrate surface coverage. A thin amorphous carbon shell surrounding the crystallites and carbon inclusions was observed, likely originating from the organic solvent. X-ray photoemission spectroscopy analysis indicated high-purity Te films with minimal surface oxidation. The small chlorine signal suggested a near-complete precursor reaction and the efficient purging of byproducts. This novel sALD approach presents a promising method for depositing Te on various surfaces under mild conditions.

## Abstract 2

This study explores the deposition of tellurium (Te) on silicon/silicon nitride substrates using solution atomic layer deposition (sALD) at ambient temperature. Utilizing TeCl_4_ and (TES)_2_Te as precursors, the process optimizes growth parameters to achieve high-purity, crystalline Te films with needle-like structures. The method shows potential for mild-condition Te deposition on diverse surfaces.

## 1 Introduction

Compounds containing chalcogen elements (sulfur (S), selenium (Se), and tellurium (Te)) from the sixth main group of the periodic table exhibit fascinating physical and chemical properties. A well-known thermoelectric material, Bi_2_Te_3_, has been extensively studied for converting waste heat into electrical energy. Since its discovery in the 1960s, the efficiency of Bi_2_Te_3_ has been continuously improved by applying various methods, such as nanostructuring or surface modification.^1,2^ Beyond these compounds, single-phase materials also exhibit intriguing properties worthy of investigation. Tellurium, a one-phase material, is particularly interesting due to its unique helical chain structure and anisotropic properties. It exhibits a combination of metallic and semiconducting behavior, with high electrical and thermal conductivity along the chain direction but poor conductivity perpendicular to the chains. Furthermore, tellurium has a tunable bandgap ranging from 0.31 eV (bulk) to 1.04 eV (monolayer), making it a promising candidate for optoelectronic and thermoelectric applications.^3−6^

However, tellurium’s scarcity, primarily obtained as a byproduct of copper refining at low concentrations (around 1 ppm in copper ore), necessitates optimizing its usage. Depositing thin films of tellurium allows for a more efficient utilization of its limited supply.

Although various deposition techniques, such as radiofrequency (RF) reactive sputtering, chemical vapor deposition (CVD), and atomic layer deposition (ALD), have been employed to obtain tellurium films, each method has its advantages and limitations. The choice of deposition technique and control of deposition parameters are crucial in determining the films’ structural, optical, and electrical properties.^7−11^

This work employs a relatively new approach termed solution Atomic Layer Deposition (sALD). ALD is based on the sequential exposure of a substrate to gas-phase precursor molecules that possess self-limiting surface chemistry. sALD transfers the principles of ALD from the gas phase to liquid processing, allowing the deposition of materials on heat-sensitive substrates such as polymers, ionic solids, and metal–organic frameworks.^12−15^ The precursor combination used in this work (TeCl_4_ + (TES)_2_Te) could not be utilized in gas-phase ALD due to the insufficient vapor pressure of TeCl_4_.

## 2 Experimental Section

### 2.1 Tellurium Deposition

**Figure 1:**
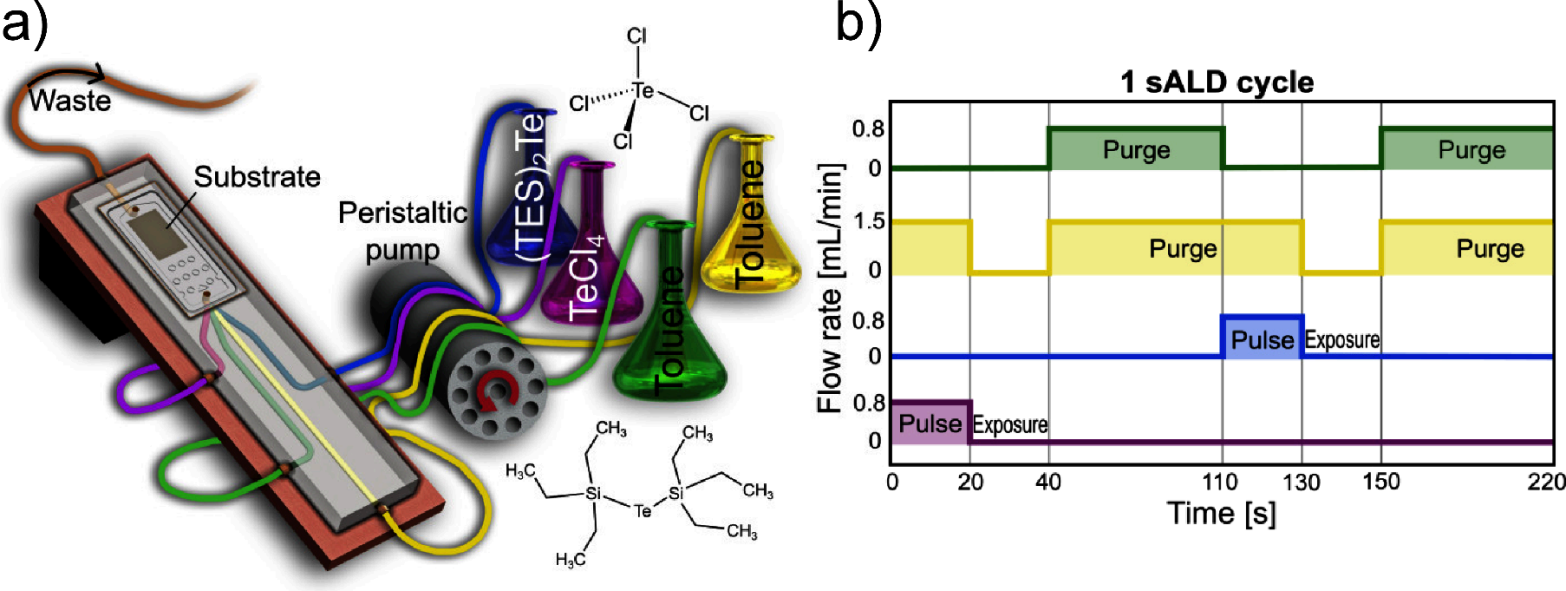
Experimental setup for deposition of tellurium on substrates showing the precursors (a) and related flow rates (b).

The deposition of tellurium was performed in a stainless steel solution atomic layer deposition (sALD) reaction chamber at ambient temperature, as shown schematically in Figure 1a. The precursors were prepared within an argon-filled glovebox. Precursor A consisted of a 1 mM solution of tellurium tetrachloride (TeCl_4_, Sigma-Aldrich, 99%), while precursor B comprised a solution of bis(triethylsilyl-) telluride ((TES)_2_Te, Dock/Chemicals, ≤100%) dissolved in Toluene. These prepared precursors were stored in Schlenk flasks, connecting them to the Schlenk line outside the glovebox to conduct the experiment under a nitrogen atmosphere. Toluene was utilized as a precursor and purge solvent, having been distilled and dried over a sodium/potassium (Na/K) alloy in a nitrogen environment.

The sequence of steps in a single sALD cycle, illustrating the flow rates of precursors and purge solvents along with the corresponding pulse, exposure, and purge durations, is depicted in Figure 1b. During an ALD cycle, the growth of the thin film is self-limiting to a single atomic layer. This arises from the fundamental property of ALD, which is that the two precursor species do not undergo reactions within the chamber. Instead, the precursors react sequentially through self-terminating surface reactions with the substrate, forming a single monolayer per cycle.

For wafer preparation, 10 × 8 mm^2^ pieces were cut from a silicon wafer coated with a 150 nm thick silicon nitride layer (Si_3_N_4_). Initially, these wafers were cleaned in an ultrasonic bath at room temperature for 10 min in isopropanol. Before the commencement of the experiment, the wafer surfaces were activated with ozone gas for 10 min using an UV Ozone Cleaner from Ossila. Following this preparation, the wafers were promptly placed in the chamber to prevent contamination. The reaction chamber was connected via polytetrafluoroethylene (PTFE) tubes to the Schlenk flasks containing the precursors and solvents and via flexible elastomeric tubes to the peristaltic pump. The wafers were stored inside the chamber, which was covered with a glass plate.

The experimental parameters are controlled by a self-written program, which allows for setting and controlling pulse, purge, and exposure times as well as the flow rate and tube diameter, during a given number of cycles.

**Figure 2:**
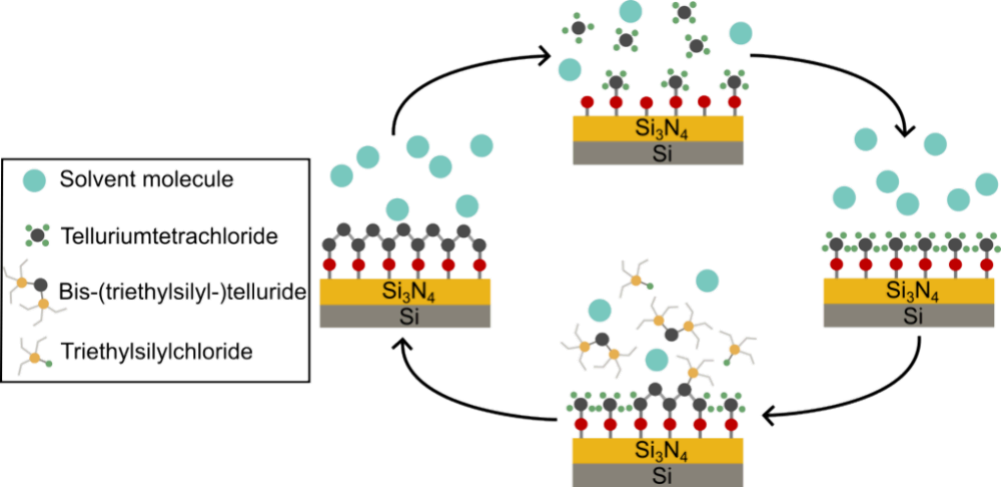
Schematic depiction of one sALD cycle showing the adsorption of precursors at the functional groups on the surface of the substrate.

The solution ALD experiment begins with a pulse of precursor A (TeCl_4_). Following the pulse step, an exposure step allows the reaction between the first precursor and substrate surface. The exposure time was set to 20 s for all experiments. This is succeeded by a purge step, during which pure toluene is pumped through the chamber to remove unreacted precursors and byproducts. The process is then repeated with precursor B ((TES)_2_Te). Specifically, the second precursor is pulsed, followed by another exposure step for the surface-precursor reaction and then another purge step. These steps constitute one complete cycle and are schematically represented in Figure 2. Multiple cycles are repeated throughout the experiment, which is conducted at room temperature. Both precursors (A and B) and one of the toluene solvents are delivered at a flow rate of 0.8 mL/min, while the second toluene solvent is delivered at a flow rate of 1.5 mL/min, as illustrated in Figure 1b).

### 2.2 Characterization of Deposited Tellurium

The crystallinity and composition of deposited the Te-sample was characterized using X-ray diffraction (XRD, with Co Kα radiation, D8 advance, Germany). The morphology was analyzed by using field emission scanning electron microscopy (FESEM, LEO1530-ZEISS FESEM). The EDX mapping was generated using a field emission scanning electron microscope (FESEM, Sigma300-ZEISS FESEM) equipped with an EDS detector. For structural and compositional TEM investigations, a holey carbon TEM grid was coated simultaneously with the silicon wafer samples. TEM and STEM observation was conducted in a TECNAI G20 at 200 kV acceleration voltage equipped with an Oxford Instruments EDX detector. X-ray photoemission spectroscopy (XPS) was performed with an ESCALAB Xi+ by Thermo Scientific XR6 monochromated Al Kα source (*h*ν = 1486.6 eV) and a pass energy of 20 eV. The growth per cycle (GPC) of the samples was measured by using an ellipsometer (Sentech Instrument GmbH). The measured shift in wavelength before and after the experiment was correlated to growth per cycle.

## 3 Results and Discussion

**Figure 3:**
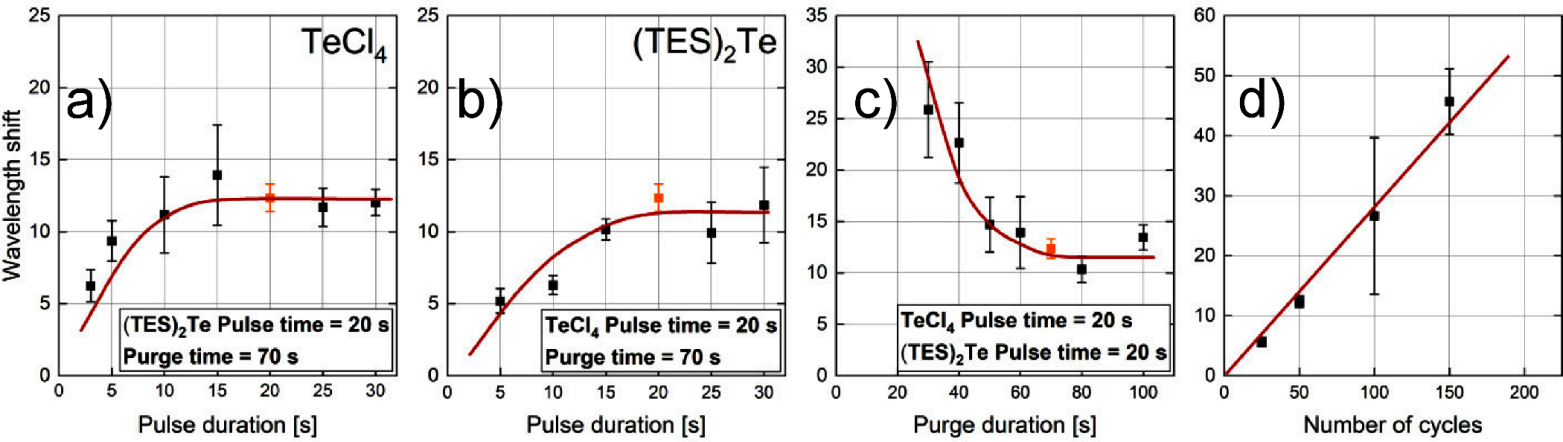
(a) Pulse-variation study of the TeCl_4_ precursor, (b) pulse-variation study of the (TES)_2_Te precursor, (c) variation study of the purge time, and (d) thickness growth study. Pulse and purge times chosen as optimum are marked in orange.

Figure 3a shows the variation study of precursor A (TeCl_4_). The pulse duration of precursor B ((TES)_2_Te) and purge duration were constant at 20 s. At pulse durations below 15 s, the wavelength shift decreases, indicating decreasing growth per cycle. Using a pulse length of 15 s or less provides not enough precursor, and therefore, the growth per cycle is reduced. A plateau arises above 15 s, which shows the self-limiting growth per cycle where all active sites (e.g., −H, −OH) at the surface reacted with precursor molecules. A further increase in the pulse duration will not have an effect on the thickness growth, and therefore, the optimized pulse duration for precursor A (TeCl_4_) was set to 20 s. Figure 3b shows the variation study of precursor B ((TES)_2_Te). Pulse and purge times of precursor A were held constant at 20 and 70 s, respectively. The wavelength shift increases until a pulse duration of 20 s, which is related to an increase in GPC. Since higher pulse durations lead to the formation of a plateau, the pulse duration for precursor B was set to 20 s, as well. Figure 3c shows the variation study of the purge duration. The pulse times for precursors A and B were maintained at a constant value of 20 s. The purge duration was systematically varied between 30 and 100 s to identify a purge time sufficiently long to remove excess precursor molecules and byproducts from the reaction chamber, thereby preventing undesired coreactions between the two precursors. Concurrently, an excessively prolonged purge duration was avoided to minimize solvent consumption and maintain an efficient process throughput. The wavelength shift decreases until 70 s, which can be attributed to a decrease in the GPC. A purge time below 70 s did not remove the excess precursor from the chamber. At a 70 s purge duration, the limitation of the growth is reached, and a plateau is formed. Figure 3d shows the thickness growth study where the number of cycles was varied. Pulse and purge duration were kept constant at their optimized parameters (20 and 70 s, respectively). With increasing cycle number, the wavelength shift increases linearly, which shows the growth limitation per cycle.

**Figure 4:**
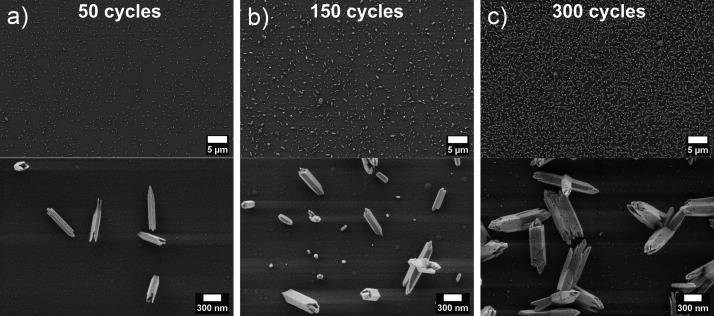
SEM top-view images of Te samples on Si_3_N_4_ substrates with optimized pulse and exposure times and varying cycle numbers: (a) 50, (b) 150, and (c) 300 cycles.

Figure 4 illustrates the morphological evolution of the samples as a function of the deposition cycle number (50, 150, and 300 cycles). As the number of cycle increases, greater surface coverage of the substrate is observed, accompanied by an enlargement of the tellurium crystals. The width of the crystals is around 100 nm for the sample with 50 cycles and increases to up to 500 nm for the sample with 300 cycles. The latter phenomenon can be attributed to the enhanced availability of precursors during the extended deposition process. The crystals exhibit a radial growth in all directions, resulting in incomplete surface coverage even after 300 cycles.

**Figure 5:**
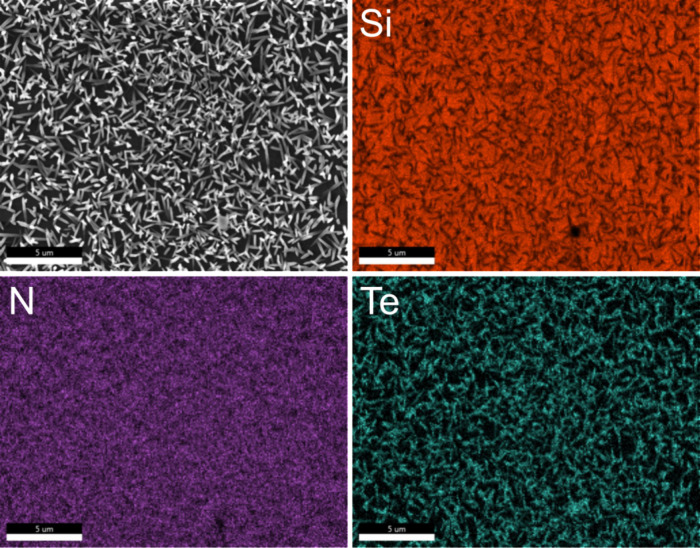
Energy-dispersive X-ray mapping of Te after 300 cycles on a silicon/silicon nitride (Si/Si_3_N_4_) substrate clearly differentiates the elemental distribution of silicon (Si), nitrogen (N), and tellurium (Te).

Figure 5 presents the energy-dispersive X-ray (EDX) mapping of a Te-sample after 300 sALD cycles on a silicon/silicon nitride (Si/Si_3_N_4_) substrate. In addition to the signals for silicon (Si) and nitrogen (N), the tellurium signal is distinctly confined to the needle-like morphology of the crystallites previously observed in the top-view scanning electron microscopy (SEM) images in Figure 4. The substrate surfaces remain uncovered, consistent with prior observations.

**Figure 6:**
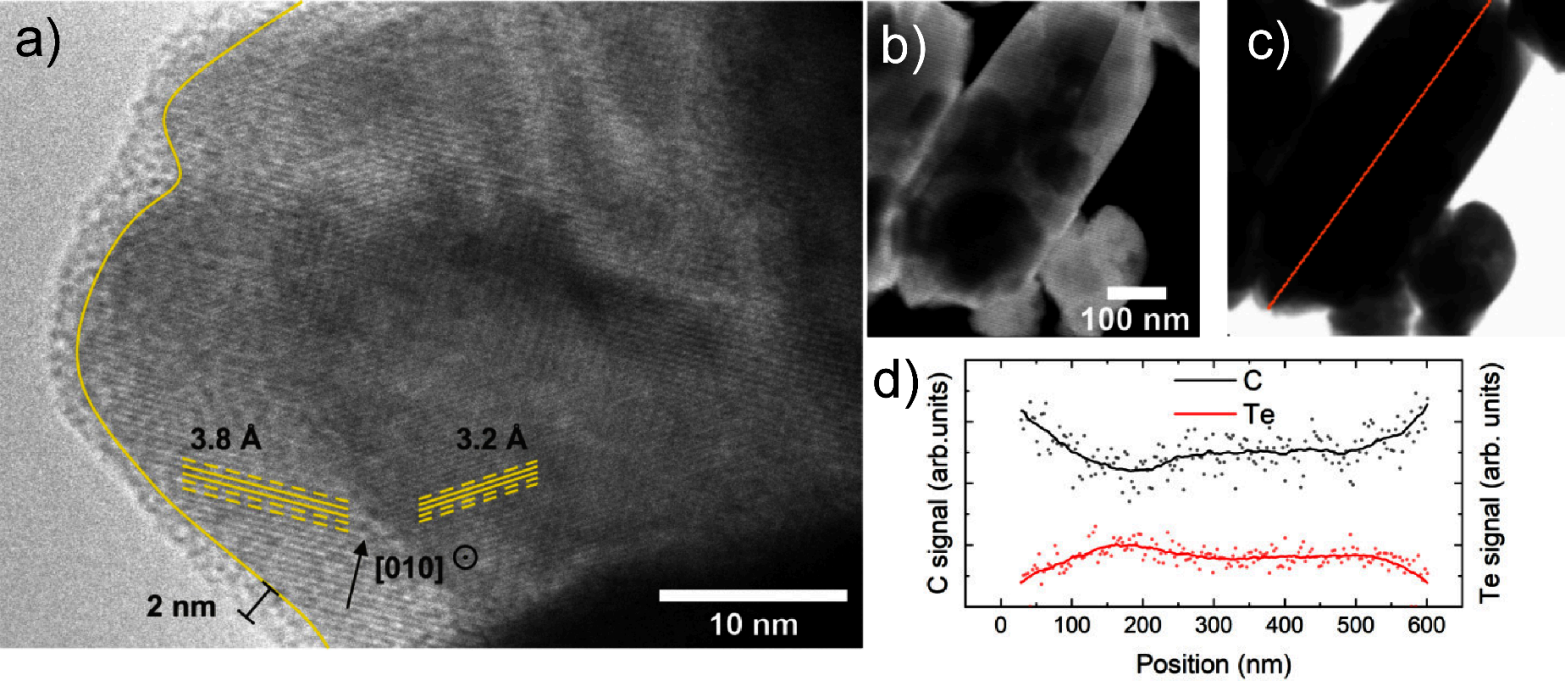
(a) TEM image of a Te-needle showing crystalline Te grains and an amorphous shell. (b) Dark field and (c) bright field STEM images of a Te-needle. (d) EDX compositional profile showing Te and C signal intensity through a Te-needle following the line depicted in (c).

Transmission electron microscopy (TEM) was employed to investigate the nanostructure of the samples. Figure 6 shows a high-resolution TEM image of a Te-needle free-standing over the edge of a hole in the carbon grid. The interference created by crystallographic planes, highlighted by the yellow lines, demonstrates that the Te needles are polycrystalline, each crystallite having a well-ordered structure. The period of the highlighted interference patterns, approximately 3.2 and 3.8 Å, lies close to projections along [010] and perpendicular to [010] of the Te lattice, respectively.^16,17^ Surrounding the Te particle is observed, an amorphous region with a thickness of approximately 2 nm is observed. This can be attributed to an organic capping layer arising from organic molecules used during the deposition procedure. Scanning transmission electron microscopy (STEM) images (Figure 6b–d) show fluctuating density across the needles, with a globular texture. This coincides with the apparent cracks or otherwise open pore structures seen in the SEM images (Figure 4c at the bottom). An EDX line scan across a Te-needle showed fluctuating proportions of the Te and C signals, pointing out the presence of organic material within the needles, most likely originated from residual solvent during synthesis.

**Figure 7:**
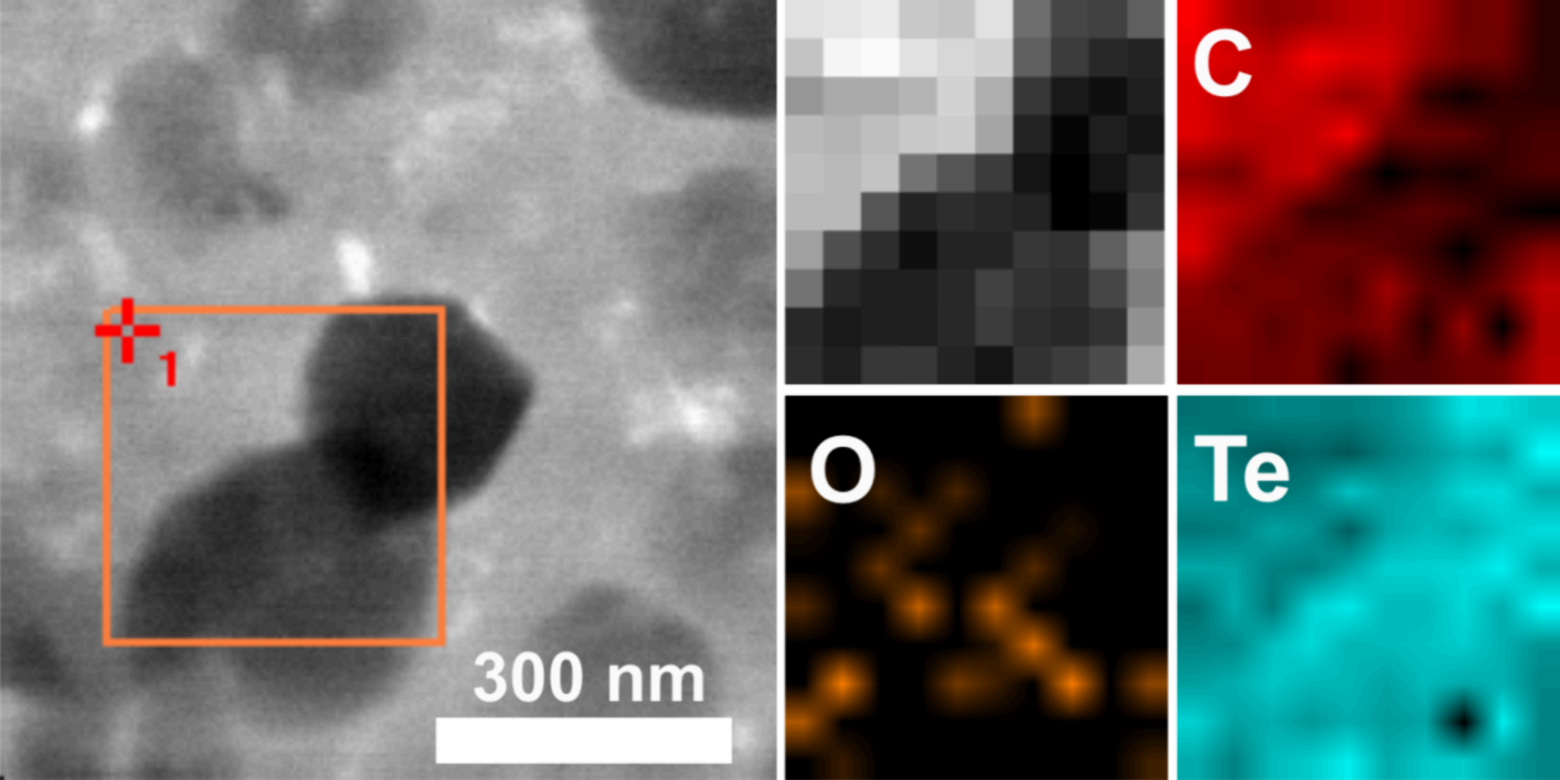
STEM bright field image of a Te layer deposited on a C-coated TEM grid. On the right side, Te, C, and O EDX signal intensity maps collected from the indicated square area. Grayscale coarse mosaic image indicates positions where EDX data were collected.

The Te-coated TEM grid was largely covered in what appeared to be a granular material with denser objects of about 200 nm in size. Once again, this feature coincides with the granular texture observed in SEM images at the surface of the coated Si/Si_3_N_4_ wafers (Figure 4a–c bottom). Figure 7 shows a composition map around a dense grain, indicating that the denser area is a Te-rich grain.

**Figure 8:**
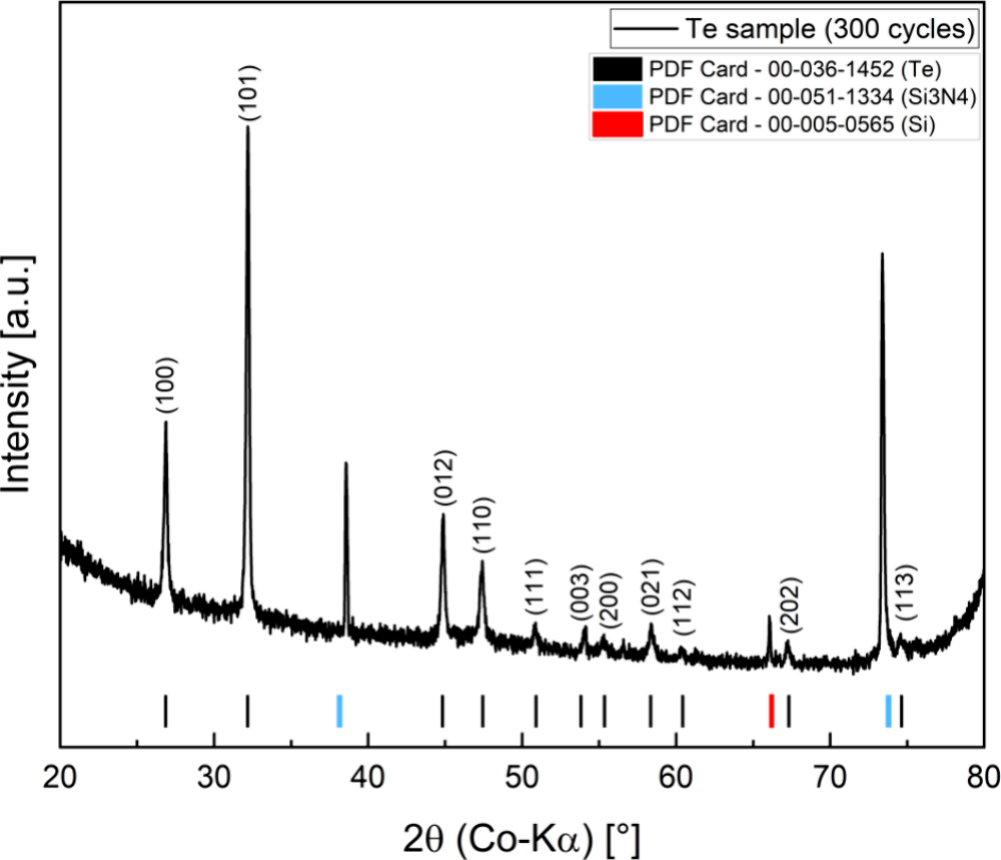
XRD of a sample with 300 cycles of Te on a Si/Si_3_N_4_ substrate.

Figure 8 shows the X-ray diffractogram (XRD) of a sample with 300 cycles of tellurium on a Si/Si_3_N_4_ substrate. The main phase can be assigned to tellurium (PDF Card #00-036-1452), and the reflexes are sharp, which indicate a high degree of crystallinity. Besides reflexes from the substrate (Si and Si_3_N_4_), no further phases were observed.

**Figure 9:**
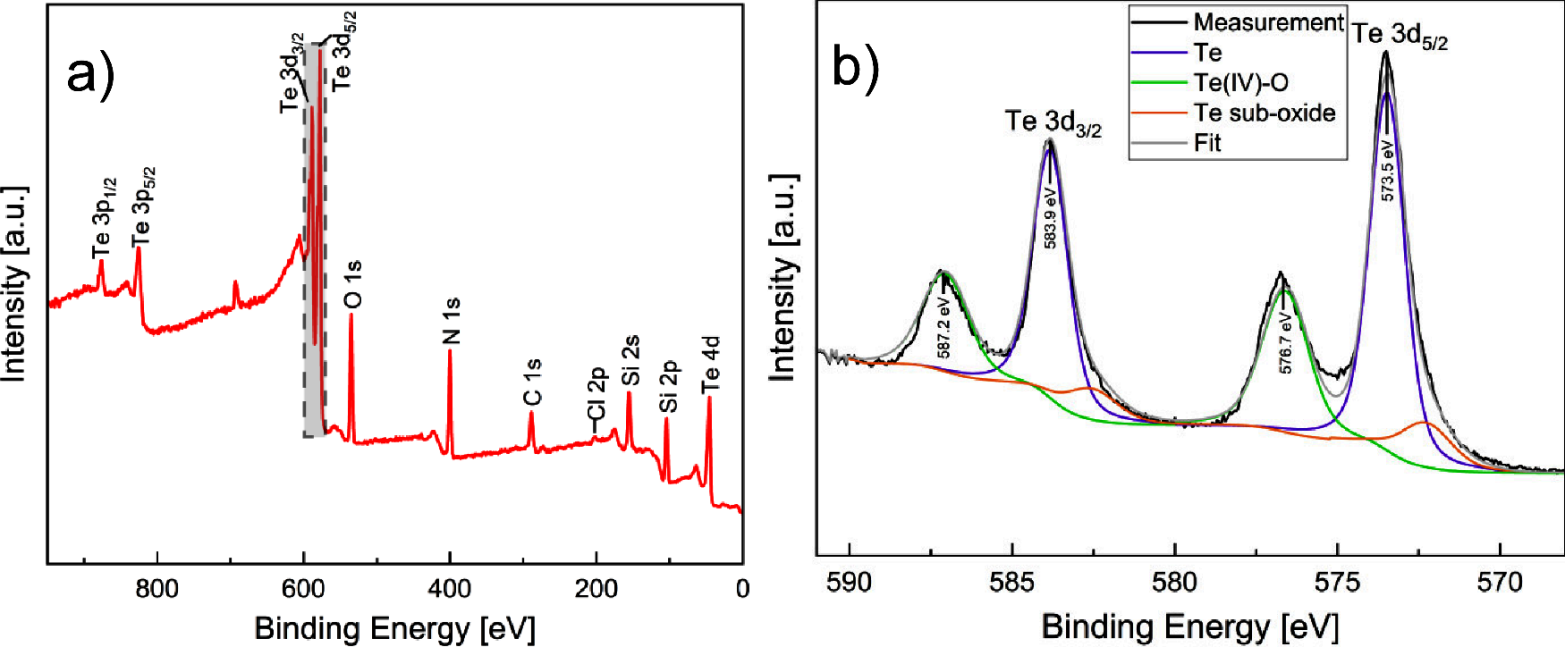
(a) XPS survey spectrum of 300 cycles of Te on a Si/Si_3_N_4_ substrate. (b) High-resolution Te 3d_3/2_ and 3d_5/2_ spectrum.

Figure 9a presents the XPS survey spectrum of a tellurium (Te) sample deposited over 300 cycles on a Si_3_N_4_ substrate. The spectrum distinctly identifies the characteristic peaks of tellurium (3d_3/2_ and 3d_5/2_). Additionally, the spectrum reveals peaks corresponding to oxygen, nitrogen, carbon, chlorine, and silicon. These extraneous peaks are attributed to the specific precursors employed during the deposition. Chlorine and carbon peaks could be explained by the precursor TeCl_4_ and (TES)_2_Te, respectively. The amount of Cl is around 2.4 atom %, while the C content with 20.9 atom % is much higher (cf. Table 1). This finding agrees with the TEM results showing carbon inclusions and surface contamination (cf. Figure 6). Silicon and nitrogen peaks are attributed to the substrate. The oxygen contained in the sample indicates oxidation of tellurium during air handling of the samples. Figure 9b shows a high-resolution spectrum of Te 3d_3/2_ and Te 3d_5/2_. Besides the peaks which were assigned to pure tellurium there are some peaks, which also indicate the formation of Te-suboxides.^18,19^

**Table 1:** Compositional Analysis Obtained from the XPS Survey Scan of a Te-Sample Deposited via 300 Cycles on a Si_3_N_4_ Substrate

name	peak BE	height [CPS]	FWHM [eV]	atomic %
Si 2p	104.24	59897.23	3.597	25.22
Cl 2p3	201.97	5148.90	6.227	2.44
C 1s	288.93	36694.02	4.477	20.90
N 1s	400.14	99608.37	3.282	24.42
O 1s	535.03	121940.53	3.640	20.38
Te 3d	578.17	304860.83	3.432	6.64

Taken together, these results are consistent with the reaction pathway described in eq 1. This mechanism can be rationalized by considering the higher affinity of chlorine for silicon compared to tellurium, which is attributed to silicon’s lower electronegativity. The XPS survey scan presented in Figure 9a corroborates this hypothesis, revealing a negligible chlorine signal, suggesting the chlorine-containing precursor’s near-complete consumption and the subsequent purging of (TES)Cl. This observation provides strong evidence for the proposed reaction mechanism and the efficient removal of the byproducts.
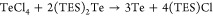
1

## 4 Conclusions

In this study, we developed a novel method for depositing elemental tellurium under ambient conditions using solution ALD using (TES)_2_Te and TeCl_4_ as precursors and toluene as the solvent. The samples, analyzed via electron microscopy, exhibited needle-like growth with high purity and crystallinity, as confirmed by XRD. Due to the radial growth of the tellurium crystals, complete surface coverage of the substrates was not achieved, even at higher cycle numbers. To improve the film growth, a surface pretreatment with small organometallic molecules could enhance nucleation by increasing the available functional groups at the surface. XPS analysis revealed the presence of a small fraction of additional elements originating from the precursors. This new method is highly interesting for depositing tellurium on a broad range of substrates and with a low thermal budget.
